# Assembly of Plasmonic and Magnetic Nanoparticles with Fluorescent Silica Shell Layer for Tri-functional SERS-Magnetic-Fluorescence Probes and Its Bioapplications

**DOI:** 10.1038/s41598-018-32044-7

**Published:** 2018-09-17

**Authors:** Hyung-Mo Kim, Dong-Min Kim, Cheolhwan Jeong, So Yeon Park, Myeong Geun Cha, Yuna Ha, Dahye Jang, San Kyeong, Xuan-Hung Pham, Eunil Hahm, Sang Hun Lee, Dae Hong Jeong, Yoon-Sik Lee, Dong-Eun Kim, Bong-Hyun Jun

**Affiliations:** 1 Department of Bioscience and Biotechnology, Konkuk University, Seoul, 05029 Republic of Korea; 2 School of Chemical and Biological Engineering, Seoul National University, Seoul, 08826 Republic of Korea; 3 Department of Chemistry Education, Seoul National University, Seoul, 08826 Republic of Korea

## Abstract

In this study, we report on the fabrication of multilayered tri-functional magnetic-SERS-fluorescence nanoprobes (MF-SERS particles) containing clustered superparamagnetic Fe_3_O_4_ nanoparticles (NPs), silver NPs, and a fluorescent silica layer. The MF-SERS particles exhibited strong SERS signals from the silver NPs as well as both superparamagnetism and fluorescence. MF–SERS particles were uptaken by cells, allowing successful separation using an external magnetic field. SERS and fluorescence signals could be detected from the NP-containing cells, and CD44 antibody-conjugated MF-SERS particles selectively targeted MDA-MB-231 cells. Based on these properties, MF-SERS particles proved to be a useful nanoprobe for multiplex detection and separation of cancer cells.

## Introduction

Multifunctional nanomaterials, which possess several different functionalities within a single nanoscale material, have recently drawn great attention for their potential applications in biological systems^1,2^. Designing the structure and components of the nanomaterials is one of the most important aspects of their fabrication, as their functions can be maximized by using customized combinations of materials. Among various functional properties, the integration of optical and magnetic properties seems to be a promising combination for cell separation and multiplex cell imaging^3–5^.

There has been growing interest in the use of optical tagging for investigation of the complex interplay of biomolecules. Among the optical tagging methods, fluorescence-based materials have been most widely used owing to their simple and broad encoding process, ease of detection, and compatibility with a variety of biochemical functions^6–10^. However, one of their most critical problems can be their broad emission profiles, which can place limits on multiplex detection^11,12^.

Nanostructures of noble metals such as gold and silver exhibit a phenomenon known as surface-enhanced Raman scattering (SERS), in which the scattering cross sections of adsorbed molecules are dramatically increased^13–15^. SERS can be used as a tagging method by combining it with Raman label compounds (RLCs). Because SERS signals have narrow bands with minimal spectral overlap, SERS can be used as a useful tool for multiplex detection^16–18^. SERS signals are commonly obtained by placing RLCs on plasmonic nanoparticles (NPs). The molecules trapped in the gaps between NPs, known as “hot spots”, can exhibit Raman signals that are several orders of magnitude more intense than those from other molecules^19,20^. Thus, assembling large amounts of Ag NPs on a backbone structure such as silica could drastically enhance the SERS signal due to the generation of hot spots, while also providing an easy-to-handle assembled nanostructure. Silica NPs have several advantages as backbones for such assembled nanostructures, such as ease of fabrication and surface modification and high stability. Recently, our group reported that Ag NPs assembled on a silica surface formed a bumpy structure, resulting in enhanced SERS intensity that can be detected from a single NP^21–23^.

Superparamagnetic NPs have attracted widespread attention owing to their lack of magnetic remanence field, which can prevent NPs from agglomerating after an external magnetic field is removed^24–26^. In particular, superparamagnetic Fe_3_O_4_ NPs have been focused for biomedical applications because of their strong saturated magnetization, non-toxicity and biocompatibility, as well as their superparamagnetic nature. This advantage is size-dependent and occurs when the size of nanoparticles is 10 to 20 nm. Common superparamagnetic NPs include γ-Fe_2_O_3_ (maghemite), Fe_3_O_4_ (magnetite) and α-Fe_2_O_3_ (hermatite)^27,28^. They are small with a core ranging from 10 to 100 nm in diameter. Their superparamagnetic properties are exhibited by mixed oxides of iron. Transition metal ions such as copper, cobalt, nickel and manganese also are in the category of superparamagnetic NPs^29^. As well, they have been used for diagnostic and therapeutic purposes. In magnetic resonance imaging (MRI), superparamagnetic NPs as magnetic resonance contrast agents have been used as targeted agents in their early stage, allowing diagnosis of progressive diseases^30–33^. For drug delivery, superparamagnetic NPs can be used for the delivery of chemotherapeutics and radiotherapeutics. However, single Fe_3_O_4_ NPs can be limited in their application due to their slow accumulation and low separation yield by magnetization. Our group recently reported a nanostructure having clustered Fe_3_O_4_ NPs on a silica core^34^. These nanostructures exhibited more rapid accumulation than single Fe_3_O_4_ NPs as well as complete separation under a magnetic field, which is useful for cell separation.

Many studies have reported on multifunctional NPs that simultaneously show the SERS and the magnetic properties, allowing magnetic isolation and detection of a target to be carried out at the same time^35,36^. However, when the dual-function magnetic-SERS NPs are used, the SERS technique still has some limitations regarding visualization or quantification of targets. In this case, fluorescence can be used as another promising optical tool to make up for the weak points of SERS. Thus, tri-functional NPs that exhibited magnetism, SERS, and fluorescence have recently been reported. However, there were still restrictions on the available RLCs due to the weak SERS signal of the material, as well as the existence of remanence magnetization even after the elimination of the external magnetic field^37,38^.

In this study, we synthesized tri-function particles (MF-SERS particles) composed of clustered Fe_3_O_4_ NPs at core part, assembled Ag NPs, and a silica shell layer containing fluorescent dye. The SERS signals from the synthesized particles were strong enough to be detected from even single asymmetric aromatic molecules, and the particles exhibited fluorescence. In addition, the particles showed superparamagnetism with a strong response to an external magnet.

## Results and Discussion

### Synthesis of MF-SERS particles

**Figure 1:**
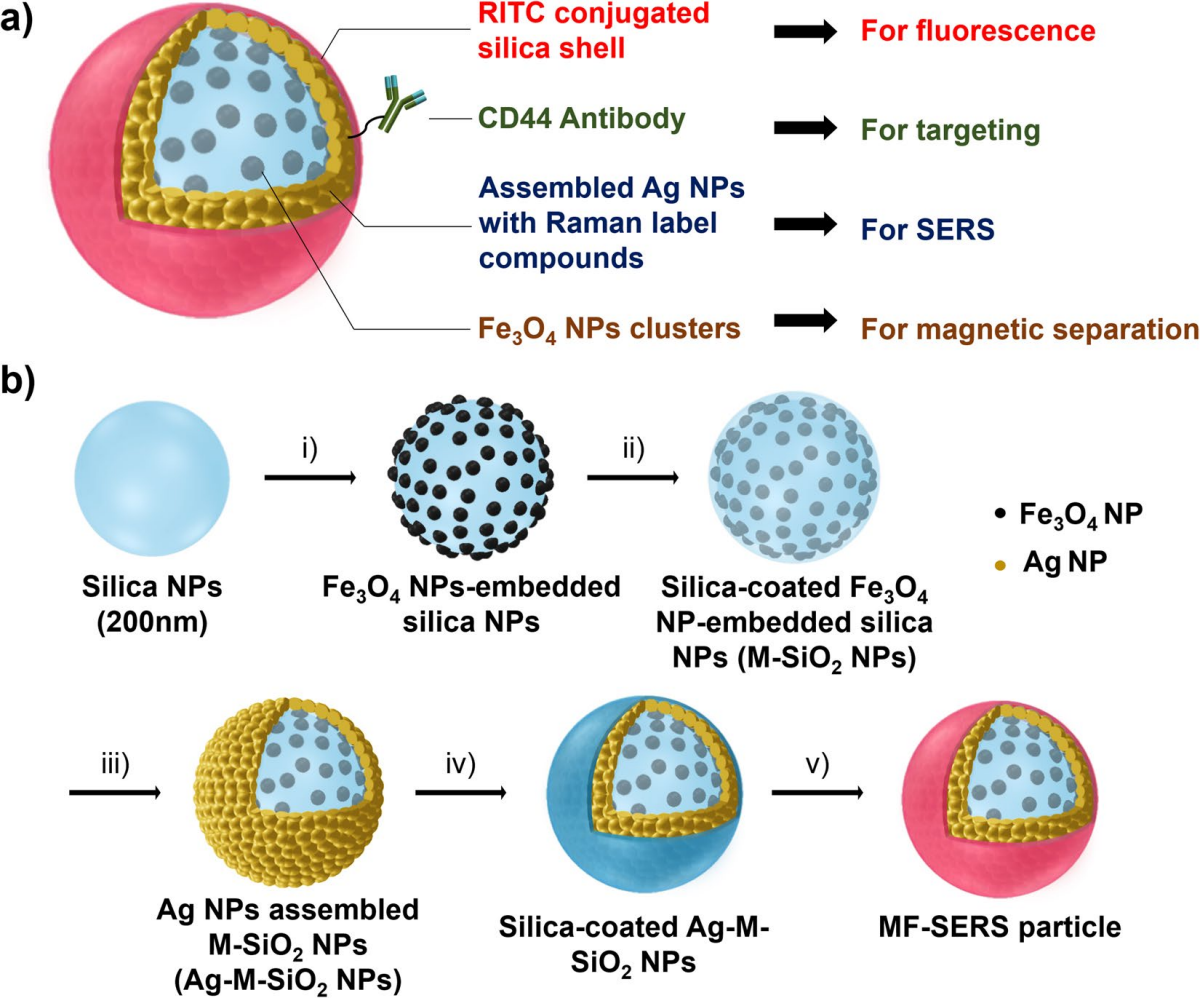
(**a**) Structure of MF-SERS particle. (**b**) Synthetic procedure of the MF-SERS particle: (i) Introduction of Fe_3_O_4_ NPs, (ii) silica coating, (iii) introduction of Ag NPs, (iv) silica coating, and (v) introduction of fluorescent shell layer.

**Figure 2:**
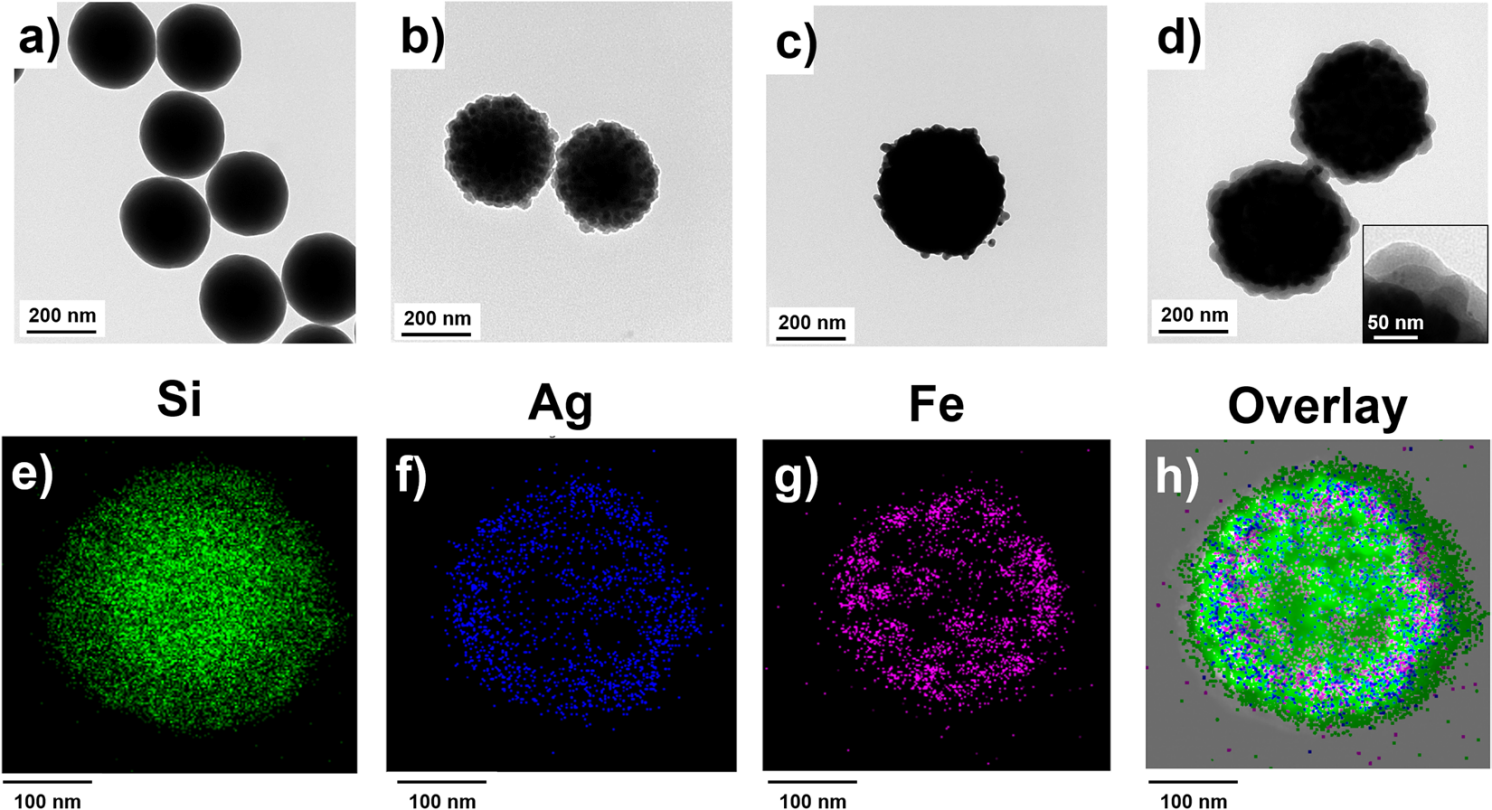
Transmission electron microscopy (TEM) images of (**a**) Silica NPs, (**b**) M-SiO_2_ NPs, (**c**) Ag-M-SiO_2_ NPs, and (**d**) MF-SERS particles. The inset shows the silica layer of MF-SERS particles. EDX mapping showing each element of MF-SERS particles including (**e**) Si atoms, (**f**) Ag atoms, (**g**) Fe atoms. (**h**) Overlay image of all elements.

The MF-SERS particles were synthesized by introducing multiple functional layers, including clustered Fe_3_O_4_ NPs, assembled Ag NPs, and a fluorescent dye-conjugated silica shell layer to provide SERS-magnetic-fluorescence tri-functionality, followed the conjugation of an antibody for biotargeting (Fig. 1a). The layers were composed of Fe_3_O_4_ NP clusters that provided a strong response to an external magnetic field due to their superparamagnetism, and assembled Ag NPs for a strong SERS signal triggered by the formation of hot spots. Figure 1b shows the synthetic procedure for preparing the MF-SERS particles. Silica NPs were used as a backbone structure to immobilize the NPs, as they can be synthesized and modified easily. The silica NPs were synthesized by the Stöber method with a narrow size distribution (250 ± 25 nm), as shown in Figs 2a and S1a^39^. When the silica NPs were synthesized, their yield was approximately 31.3% (500 mg). Amine groups were then introduced onto the silica NPs using APTS. To immobilize the superparamagnetic Fe_3_O_4_ NPs on the surface of the silica NPs, amine-functionalized silica NPs were coupled with caffeic acid to introduce catechol groups, which are known to have a strong affinity for Fe_3_O_4_ NPs^40^. We used superparamagnetic NPs with an average diameter of 18 nm. They were well dispersed and displayed a uniform size. (Fig. S2a). To confirm the magnetic properties of the Fe_3_O_4_ NPs, the field-dependent magnetization was measured at 300 K (Fig. S2b). The magnetization curve exhibited a saturated magnetization of 36 emu/g without coercivity, indicating that the Fe_3_O_4_ NPs were superparamagnetic. To avoid aggregation of the Fe_3_O_4_ NPs in the amphiphilic solvent when reacting with catechol-functionalized silica NPs, oleate-stabilized Fe_3_O_4_ NPs underwent a ligand exchange process. Oleate-stabilized Fe_3_O_4_ NPs were treated with PVP at 100 °C, and then cooled to room temperature. The resulting Fe_3_O_4_ NPs showed good dispersion in amphiphilic solvents, which confirms that the oleate ligands were replaced by PVP. The PVP-stabilized Fe_3_O_4_ NPs were then mixed with the catechol-functionalized silica NPs to immobilize the Fe_3_O_4_ NPs on the silica NPs. A silica layer with a thickness of 10 nm was then introduced onto the Fe_3_O_4_ NP-embedded silica NPs to allow further surface modification (Figs 2b and S1b), and the resulting silica-coated Fe_3_O_4_ NP-embedded silica NPs (M-SiO_2_ NPs) were successfully synthesized similar to the previous results^34,41^. And, when they were synthesized, their yield was 12.8% (5.6 mg from 4 mg of caffeic acid modified SiO_2_ with 12.5 mg of Fe_3_O_4_ NPs.).

Next, the surface of the M-SiO_2_ NPs was functionalized with thiol groups using MPTS to attract the Ag ions and aid the formation of Ag NPs^42^. Ag ions were reduced using octylamine, and enormous amounts of spherical Ag NPs (27 ± 3.2 nm) were assembled on the surface of the M-SiO_2_ NPs (Figs 2c and S1c, S3). Thiol group containing aromatic compounds were used as RLCs. The surface of the Ag NPs-assembled M-SiO_2_ NPs (Ag-M-SiO_2_ NPs) was treated the RLCs, and then coated with silica to provide chemical stability and biocompatibility. To introduce the fluorescent shell, RITC was conjugated with APTS. Finally, approximately 0.8 mg MF-SERS particles were obtained^43^. The silicate monomer-conjugated RITC was then reacted with the silica-coated Ag-M-SiO_2_ NPs. The thickness of the outer silica layer of the NPs was 17.19 ± 1.4 nm, as measured from a TEM image (Fig. 2d, inset). The synthesized fluorescent shell-coated Ag-M-SiO_2_ particles (MF-SERS particles) had uniform size distributions of 400 ± 40 nm (Fig. S1d). The chemical compositions of the MF-SERS particles were investigated by EDX. The locations of the Si, Ag, and Fe atoms in the MF-SERS particles are shown in Fig. 2e–g. In addition, the size of each element passing through the particles allowed the determination of the boundary between AgNPs and Fe_3_O_4_ (Fig. S4). These results prove that the MF-SERS particles were successfully fabricated.

### Characterization of the MF-SERS particles

**Figure 3:**
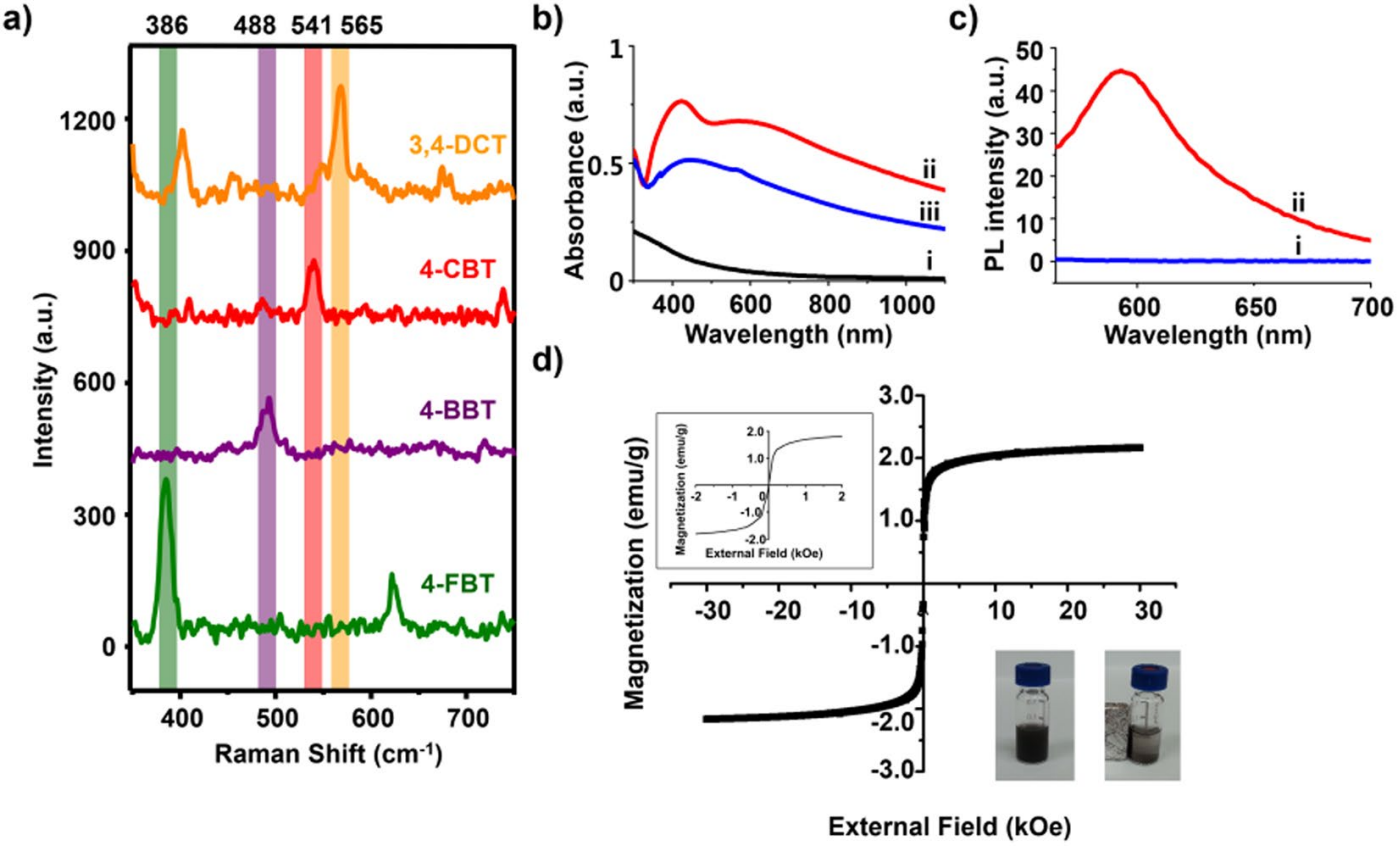
(**a**) SERS signals of MF-SERS particles. The type of Raman labeling compounds (RLCs) are 3,4-dichlorobenzenethiol (3,4-DCT), 4-chlorobenzenethiol (4-CBT), 4-bromobenzenethiol (4-BBT) and 4-fluorobenzenethiol (4-FBT). (**b**) UV spectra of NPs in each steps (i: M-SiO_2_ NPs, ii: Ag-M-SiO_2_ NPs and iii: MF-SERS particles). (**c**) Photoluminescence spectra of i: silica coated Ag-M-SiO_2_ NPs and ii: MF-SERS particles. (**d**) Hysteresis loop of the MF-SERS particles. The inset (left) shows low field region of MF-SERS particles. The inset (right) shows photographic images of the MF-SERS particles before (left) and after (right) attracted by magnet.

In order to provide MF-SERS particles with multiplex abilities, four RLCs (4-FBT, 4-CBT, 4-BBT, and 3,4-DCT) were used. SERS signals were obtained from the RLC-treated MF-SERS particles, and the unique SERS patterns of the respective molecules could be clearly distinguished by their narrow bands at 386 cm^−1^ (4-FBT), 488 cm^−1^ (4-BBT), 541 cm^−1^ (4-CBT), and 565 cm^−1^ (3,4-DCT) (Fig. 3a). Among the RLCs, even asymmetric aromatic compound (3,4-DCT), which usually gives low SERS signals, could also generate strong SERS signals due to the bumpy structure of Ag NPs layer of the MF-SERS particles. Thus, various aromatic compounds can be used as RLCs, expanding the multiplexing capability of MF-SERS particles.

The extinction spectra of NPs during every synthetic step for MF-SERS particles fabrication are shown in Fig. 3b. Compared to the absorption band of the M-SiO_2_ NPs, the Ag-M-SiO_2_ NPs showed an absorption band at *ca*. 400 nm and a broad band ranging from visible to NIR region. This was mainly due to the plasmonic property of the Ag NPs and their aggregate. This shows that the Ag NPs were well preserved after the silica shell coating step, as confirmed by TEM (Fig. 2d) and plasmonic properties of the Ag NPs were preserved during the NP’s synthesis.

We also analyzed the photoluminescence spectrum of the MF-SERS particles with a 540 nm photo-excitation (Fig. 3c). An emission band at 580 nm was observed from MF-SERS particles, corresponding to the emission band of RITC, indicating that fluorescent RITC molecule was well introduced in the silica shell layer.

To confirm the reproducibility of the MF-SERS particles, three batches of particles were synthesized, and the absorbance at 430 nm and PL intensity at 540 nm were measured (Fig. S5a). Additionally, the MF-SERS particles were consistently well dispersed in several solvents, including ethanol, PBS (pH 7.4) and cell culture media (Fig. S5b). The results indicate that MF-SERS particles are dispersed in the silica layer and are suitable for cell studies.

To confirm the magnetic properties of the MF-SERS particles, the field-dependent magnetization was measured at 300 K (Fig. 3d). The magnetization curve exhibited a saturated magnetization of 2.1 emu/g without coercivity, indicating that the MF-SERS particles were superparamagnetic. In addition, MF-SERS particles were attracted to a magnet within 10 min, which is more advantageous for cell separation than single magnetic NPs (Fig. 3d, inset).

### Cellular binding of MF-SERS particles

**Figure 4:**
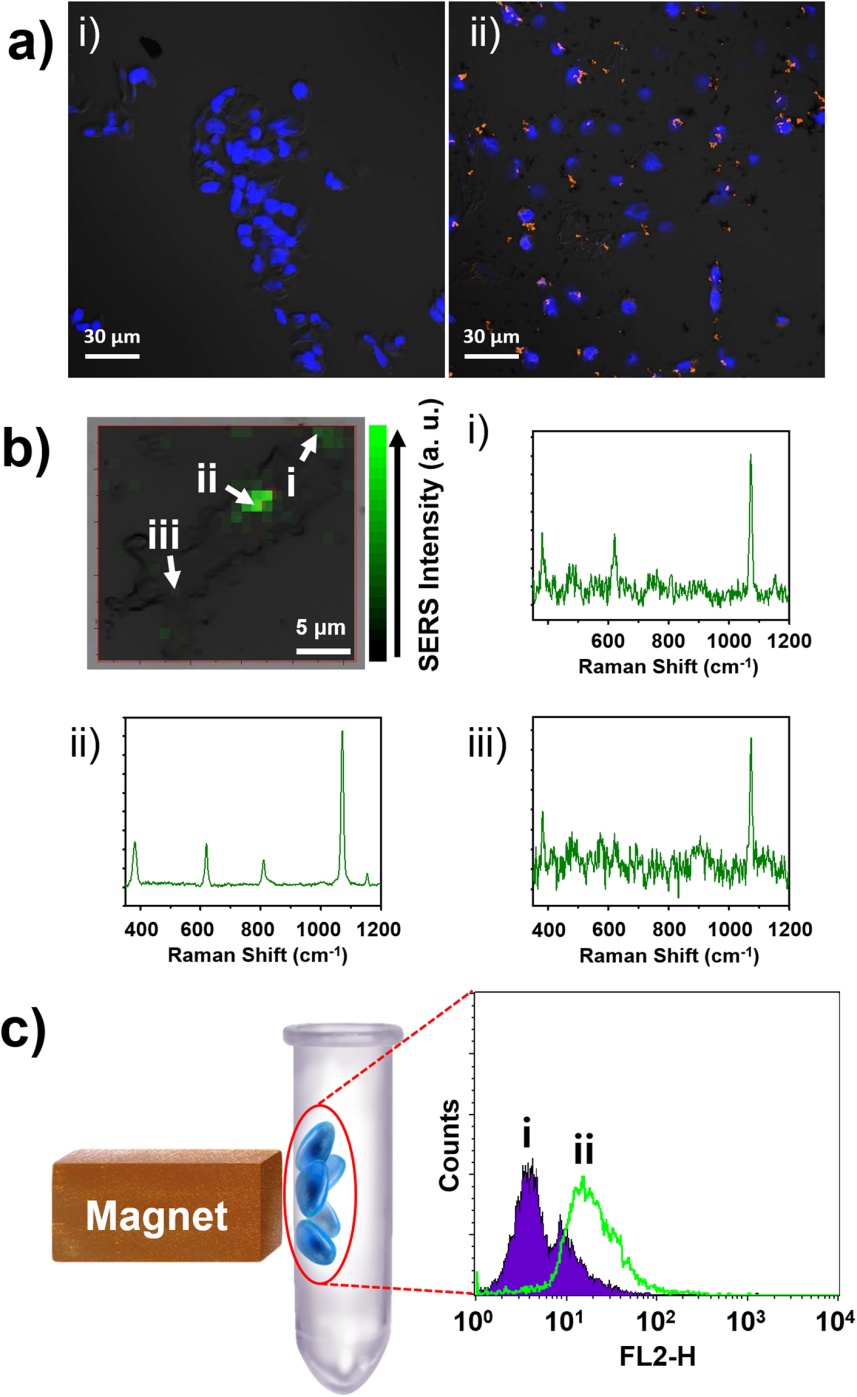
(**a**) Fluorescence microscope images of MDA-MB-231 cells incubated (i) without and (ii) with MF-SERS particles and stained with TOPRO-3 (pseudo-blue fluorescence), showing the binding of MF-SERS particles (orange fluorescence) to MDA-MB-231 cells. The microscopic images were merged with the bright field cell images which show the cell boundary. (**b**) SERS intensity map of MF-SERS particles in MDA-MB-231 cells, overlaid with the corresponding bright-field optical image. 4-FBT was used as the RLC, and the Raman spectrum of 4-FBT could be obtained from positions i, ii, and iii. (**c**) Flow cytometry analysis of MDA-MB-231 cells used as a control (Population i) and cells collected from the wall after treatment with MF-SERS particles and exposure to a magnet (Population ii).

Several studies have been reported on the interaction of NPs (~400 nm) with cells. NPs of approximately 400 nm size are known to bind to cells with about 50% being absorbed into the cell^44^. In addition, NPs with positive surface charges can have strong interactions with cells because the surface of the cells is negatively charged^45^. Thus, the surface of the MF-SERS particles was modified with APTS. The amine-functionalized MF-SERS particles (MF-SERS particles_amine_) were then incubated with MDA-MB-231 cells on a glass slide at 37 °C for 2 h. The cells with and without MF-SERS particles_amine_ were visualized by confocal microscopy to evaluate whether cellular binding or uptake had occurred (Fig. 4a). After proper washing, significant amount of orange fluorescence was observed from the MF-SERS particles_amine_ around the nuclei of the MDA-MB-231 cells (blue). In addition, when various amounts of MF-SERS particles_amine_ were treated to MDA-MB-231 cells, and the cells were analyzed by FACS, the intensities of the fluorescently-labeled cells were increased by increasing the amount of MF-SERS particles_amine_ (Fig. S6). These results suggest that the MF-SERS particles_amine_ (~400 nm) could bind to the cell surface.

SERS signals from MF-SERS particles bound cells were detected using point-by-point mapping using 660 nm laser excitation at a power of 11.8 mW with a step size of 1 µm and an exposure time of 1 s per point. The SERS map was then overlaid with the corresponding bright-field optical image, as shown in Fig. 4b. The SERS spectrum of the 4-FBT, that had been labeled as a RLC, could be obtained from regions (i), (ii), and (iii) in Fig. 4b. The SERS intensity in the SERS map was based on the height of the most intense peak of the 4-FBT spectrum, at 1075 cm^–1^. As a result, SERS signals from MF-SERS particles could be collected even from cells.

Next, we attempted to separate the MF-SERS particles bound cells using an external magnetic field. The MF-SERS particles_amine_ were mixed with MDA-MB-231 cells that were floating freely in the cell culture medium at 37 °C for 2 h. Then, a magnet was placed at the side of the cell mixture until the cells were pulled toward the magnet, as shown in Fig. 4c. The pulled down cells were collected and analyzed by fluorescence-activated cell sorting (FACS). As a result, cells with enhanced fluorescence emission were separated by FACS, as shown in Fig. 4c (population ii). Because the cells contain many MF-SERS particles, they had stronger fluorescence intensity than untreated MDA-MB-231 cells (Population i in Fig. 4c), resulting in a shift to the right in the FACS analysis. Furthermore, pure MF-SERS particles were analyzed by FACS, and the results were compared to those obtained with the MDA-MB-231 cells (Fig. S7). No particles were detected by FACS, showing that MF-SERS particles without interaction with cells during incubation period cannot be detected by FACS. Thus, these results indicate that MF-SERS particles bound cells were readily separated by external magnetic field. We also carried out a cell viability assay to evaluate the cytotoxicity of MF-SERS particles (Fig. S8). Dosages of MF-SERS particles at concentrations used in this study (from 0.1 to 10 µg/mL) showed a level of cell viability similar to the untreated group (0 µg/mL). These results indicate that there is no cytotoxicity of MF-SERS particles treated into the cell.

### Specific binding of CD44 antibody-conjugated MF-SERS particles to MDA-MB-231 cells

**Figure 5:**
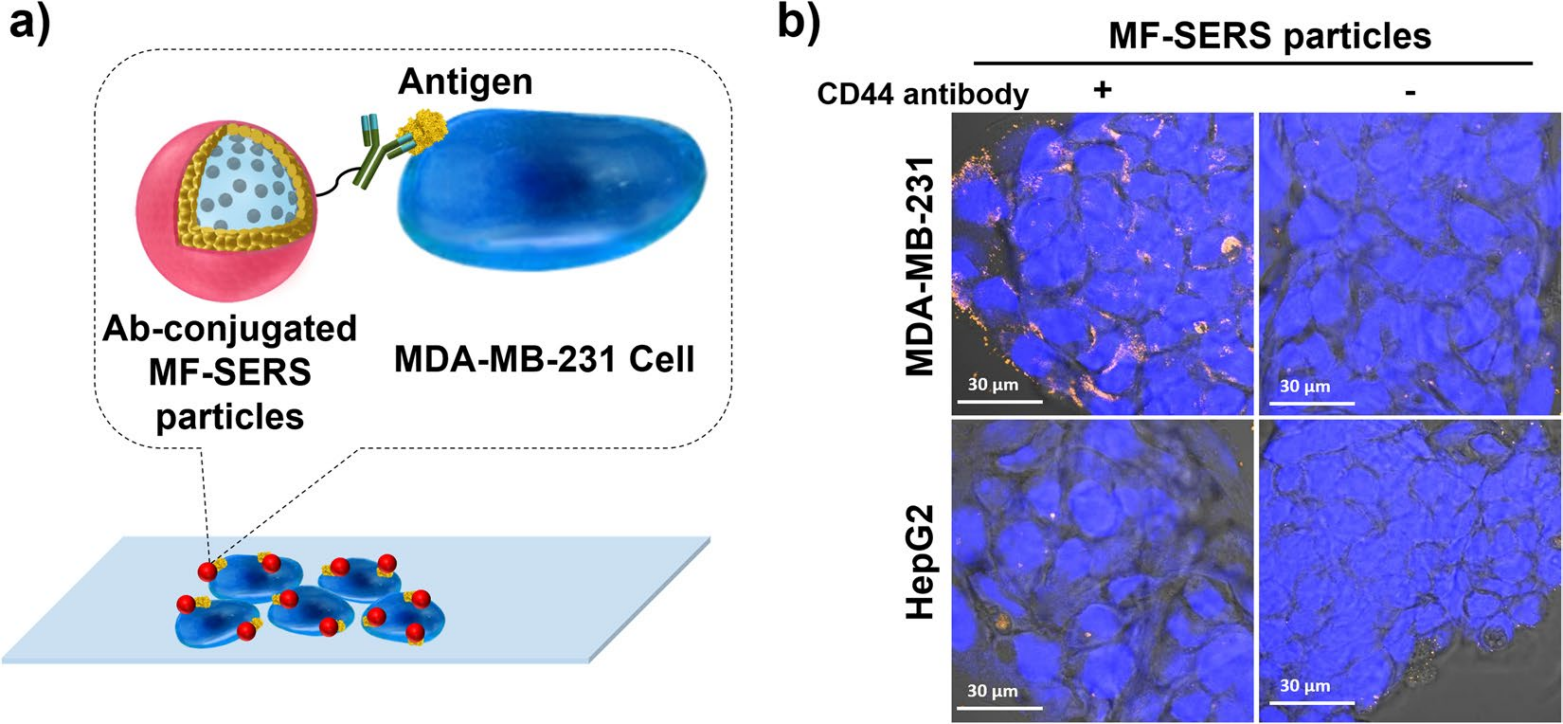
(**a**) Schematic of antigen recognition by antibody-conjugated MF-SERS particles. (**b**) Confocal fluorescence microscopy images of MDA-MB-231 cells treated with CD44 antibody-conjugated MF-SERS particles or MF-SERS particles, and HepG2 cells treated with CD44 antibody-conjugated MF-SERS particles or MF-SERS particles at 4 °C for 2 h. The orange fluorescence of the MF-SERS particles was monitored, and the nuclei were stained by TOPRO-3 to obtain pseudo-blue fluorescence. The bright field of cell images were also merged.

The antigen-specific binding of the CD44 antibody-conjugated MF-SERS particles (MF-SERS particles_Ab_) to CD44-expressing cells was investigated. First, the surface of the MF-SERS particles was modified via EDC/NHS coupling reaction in order to immobilize antibodies on the MF-SERS particles^46^. Briefly, the surface of the MF-SERS particles was functionalized with amine groups using APTS. Then, the amine groups on the surface of the MF-SERS particles were reacted with succinic anhydride to transform to carboxyl groups. The carboxyl groups were then activated by EDC/NHS for CD44 antibody coupling. After the CD44 antibody was conjugated to carboxyl groups, and the resulting MF-SERS particles_Ab_ were incubated with CD44-expressing MDA-MB-231 cells at 4 °C for 2 h. A schematic illustration of the antigen-specific binding of the MF-SERS particles with the CD44-expressing cells is shown in Fig. 5a. We examined the CD44 expression in MDA-MB-231 or HepG2 cells by immunostaining by green fluorescence (Fig. S9). The green fluorescence for the CD44 antigen was not observed in the HepG2 cells, while strong green fluorescence clearly was observed in the MDA-MB-231 cells. In the MDA-MB-231 cells, the orange fluorescence of the MF-SERS particles_Ab_ was also clearly observed at the periphery of the cells with pseudo-blue fluorescent nuclei (Fig. 5b). Additional fluorescent cell images using confocal Z-stack acquisition were also obtained to demonstrate that the location of MF-SERS particles_Ab_ appeared as orange or red fluorescence inside MDA-MB-231 cells (Fig. S10 and Movie S1 in Supplementary information). As shown in Fig. S11 and movie clips (Movies S2 and S3 in Supplementary Information), orthogonal images from XZ, YZ, and XY projections with different Z-axis clearly demonstrate that MF-SERS particles_Ab_ were internalized into the MDA-MB-231 cells. However, the orange fluorescence was rarely observed due to the absence of CD44 antibody conjugation or the CD44-negative HepG2 cells. These results prove that the MF-SERS particles_Ab_ selectively recognized the CD44 antigen in the CD44-expressing MDA-MB-231 cells.

MF-SERS particles containing magnetic, fluorescence, and SERS properties were fabricated by immobilizing superparamagnetic Fe_3_O_4_ NP clusters on silica NPs, assembling Ag NPs on them, and introducing a fluorescent silica layer. SERS signals were successfully obtained from aromatic RLCs (4-FBT, 4-CBT, 4-BBT, and 3,4-DCT) coated on the MF-SERS particles, and fluorescence signals were also obtained at the same time. The MF-SERS particles exhibited a strong response to an external magnetic field due to their superparamagnetic property. When cells were treated with the MF-SERS particles, the NP-bound cells could be separated from the others using external magnetic field and measured by FACS analysis. The characteristics of each modality of the MF-SERS particles, including fluorescence, SERS, and magnetic properties, were preserved after cellular uptake. Moreover, the MF-SERS particles could be modified with the CD44 antibody via an amide coupling reaction, and successfully targeted the CD44-positive cells. The tri-functional particles with SERS, magnetic, and fluorescent properties are expected to be useful nanoprobes for cell separation and multiplexed detection.

## Methods

### Chemical and materials

All reagents were used as received from the suppliers without further purification. Absolute ethanol (Abs. EtOH, 99.9%) was purchased from Carlo Erba. Oleate-stabilized Fe_3_O_4_ NPs were purchased from Ocean Nanotech. Tetraethyl orthosilicate (TEOS), 3-mercaptopropyltrimethoxysilane (MPTS), 3-aminopropyltriethoxysilane (APTS), caffeic acid, *N*,*N*-diisopropylethylamine (DIEA), polyvinylpyrrolidone (PVP, M_w_ 10000 or 40000), ethylene glycol (EG), silver nitrate (AgNO_3_, 99.99%), octylamine, 4-bromobenzenethiol (4-BBT), 4-chlorobenzenethiol (4-CBT), 4-fluorobenzenethiol (4-FBT), 3,4-dichlorobenzenethiol (3,4-DCT), rhodamine B isothiocyanate (RITC), *N*-hydroxysuccinimide (NHS), 4-(dimethylamino)pyridine (DMAP), and *N,N′*-diisopropylcarbodiimide (DIC) were purchased from Sigma Aldrich (St. Louis, MO, USA). (2-(1*H*-Benzotriazole-1-yl)-1,1,3,3-tetramethyluronium hexafluorophosphate (HBTU) and hydroxybenzotriazole (HOBt) were purchased from Bead Tech. Isopropyl alcohol (IPA), ethyl alcohol (EtOH, 95%), *N*,*N*-dimethylformamide (DMF), *N*-methyl-2-pyrrolidone (NMP), methylene chloride (MC), diethyl ether, and aqueous ammonium hydroxide (NH_4_OH, 27 wt.% in water) were purchased from Daejung (Siheung, Korea). Dulbecco’s modified Eagle’s medium (DMEM) with high glucose and fetal bovine serum (FBS) were purchased from HyClone Laboratories (Logan, UT, USA). The CD44 antibody was purchased from Abcam (Cambridge, MA, USA).

### Preparation of MF-SERS particles

Oleate-stabilized Fe_3_O_4_ NPs (2.5 mg in 100 μL of chloroform) and 60 mg of PVP (M_w_ = 10000) were placed in DMF/MC co-solvent (5 mL, 1:1 v/v) and heated for 18 h at 100 °C. Then, the reaction mixture was cooled to 25 °C and poured slowly into 10 mL of diethyl ether. The mixture was centrifuged (4500 rpm, 5 min) then re-dispersed in EtOH. The silica NPs were synthesized by the Stöber method. TEOS (1.6 mL) and NH_4_OH (4 mL) were added to abs. EtOH (40 mL) and stirred for 20 h at 25 °C. The reaction mixture was washed several times with EtOH by centrifuging at 7000 rpm for 15 min. To introduce amine groups onto the surface of the silica NPs, silica NPs (40 mg in 20 mL EtOH) were incubated with APTS (100 μL) and NH_4_OH (100 μL) for 18 h at 25 °C. The reaction mixture was washed several times with EtOH, and then, re-dispersed in NMP.

The amine-functionalized silica NPs (20 mg in 5 mL DMF) were mixed with caffeic acid (7.2 mg) and one equivalents of HBTU, HOBt, and DIEA, and then reacted for 3 h at 25 °C. The reaction mixture was washed several times with DMF.

The catechol-functionalized silica NPs (1 mg in 5 mL DMF) and the PVP-stabilized Fe_3_O_4_ NPs (0.4 mg in 5 mL EtOH) were mixed and sonicated for 1 h at 25 °C, and then, the reaction mixture was washed several times with EtOH.

For the silica coating, TEOS (50 μL) and NH_4_OH (100 μL) were added to the Fe_3_O_4_ NP-embedded silica NPs (1 mg in 5 mL EtOH) and reacted for 18 h at 25 °C.

For introduction of thiol groups onto the surface of the M-SiO_2_ NPs, the M-SiO_2_ NPs (1 mg in 1 mL EtOH) were mixed with MPTS (50 µL) and NH_4_OH (10 µL) and reacted for 1 h at 50 °C. The reaction mixture was washed several times with EtOH.

For introduction of Ag NPs, thiolated M-SiO_2_ NPs (1 mg in 25 µL EtOH) were poured into AgNO_3_ solution (1.25 mg in 1 ml of EG), and then 2 µL of octylamine was injected into the solution. The resulting reaction mixture (Ag-M-SiO_2_ NPs) was reacted for 1 h at 25 °C, and then washed several times with EtOH. Four compounds (3,4-DCT, 4-CBT, 4-BBT, and 4-FBT) were selected as RLCs. Ag-M-SiO_2_ NPs (0.5 mg) were mixed with each RLC solution (10 mM, 1 mL in EtOH) and reacted for 1 h at 25 °C. The reaction mixture was washed several times with IPA.

For silica coating, PVP (1 mg, M_w_ = 40000), water (60 µL), TEOS (10 µL), and NH_4_OH (10 µL) were added to the RLC-treated Ag-M-SiO_2_ NPs and reacted for 18 h at 25 °C. The silica-coated Ag-M-SiO_2_ NPs were washed several times with IPA.

For introduction of fluorescent silica shells, RITC (8 mM in 50 µL EtOH) was reacted with APTS (19.2 mM in 500 µL EtOH) for 16 h at 25 °C. A portion of the resulting solution (20 µL) was added to the silica-coated Ag-M-SiO_2_ NPs (in 2 mL IPA), along with water (400 µL), TEOS (2 µL), and NH_4_OH (10 µL). The mixture was incubated for 18 h at 25 °C, and the resulting fluorescence shell-coated Ag-M-SiO_2_ particles (MF-SERS particles) were washed several times with EtOH.

For introduction of amine groups, the MF-SERS particles (0.5 mg in 1 mL EtOH) were incubated with APTS (50 µL) and NH_4_OH (10 µL) and reacted for 1 h at 25 °C. The reaction mixture was washed several times with EtOH, and the resulting amine-functionalized MF-SERS particles (MF-SERS particles_amine_) were re-dispersed in PBS buffer solution (10 mM, pH 7.4).

For introduction of antibodies onto the MF-SERS particles, MF-SERS particles_amine_ were re-dispersed in NMP (0.5 mL) and reacted with succinic anhydride (1.75 mg) and DIEA (3.05 µL) for 2 h at 25 °C. The reaction mixture was washed several times with DMF. The resulting carboxyl group-ended MF-SERS particles in 200 µL DMF were incubated with NHS (40 mg), DMAP (4.2 mg), and DIC (54 µL) and reacted for 2 h at 25 °C. The reaction mixture was washed several times with PBS buffer solution at 4 °C. CD44 antibody (25 µg) was added to the NHS activated MF-SERS particles (in 200 µL PBS buffer solution) and reacted for 1 h at 25 °C. The reaction mixture was washed with PBS buffer solution containing 0.1 wt.% TWEEN 20, and the antibody-conjugated MF-SERS particles (MF-SERS particles_Ab_) were then re-dispersed in PBS buffer solution.

### Characterization of the MF-SERS particles

Transmission electron microscope (TEM) images of NPs were obtained using a Carl Zeiss LIBRA 120 (Oberkochen, Germany), and a JEOL JEM-3000F (Tokyo, Japan) was used for energy-dispersive X-ray spectroscopy (EDX) mapping imaging analysis. SERS measurements were performed using a micro-Raman system (LabRam 300, JY-Horiba). Extinction properties of NP samples were analyzed using a UV/vis spectrophotometer (Mecasys OPTIZEN POP, Daejeon, Korea). Photoluminescence intensities were obtained using a fluorescence spectrophotometer (Model Cary Eclipse, Agilent Technologies, Santa Clara, CA, USA). Field-dependent magnetization of dried MF-SERS particles was measured using a PPMS-14 (Quantum Design, USA). Fluorescence microscopic images were obtained using a confocal laser scanning microscope (Olympus FV-1000 spectral, Tokyo, Japan).

### Cell culture and internalization of MF-SERS particles

MDA-MB-231 cells (breast cancer epithelial cell line, purchased from American Type Culture Collection, ATCC HTB-26) and HepG2 cells (liver epithelial cell line, purchased from ATCC, ATCC HB-8065) were cultured in Dulbecco’s modified Eagle’s medium with high glucose (HyClone Laboratories, Logan, UT, USA) supplemented with 10% fetal bovine serum (HyClone Laboratories) and 100 U/mL of penicillin (Welgene, Daegu, Korea) at 37 °C in humidified air containing 5% CO_2_. To determine the cellular binding of MF-SERS particles_amine_ to the cells, MDA-MB-231 cells were seeded onto a 24-well plate with cover glass (Paul Marienfeld GmbH, Lauda-Königshofen, Germany) at a density of 2.0 × 10^5^ cells and incubated at 37 °C. After 16 h of incubation, the cover glass was blocked with 3% bovine serum albumin (BSA) in PBS at room temperature for 30 min. After washing with PBS twice, 500 μL of media containing 0.25 mg/mL of MF-SERS particles_amine_ was added to the cells and incubated again at 37 °C for 2 h. The cells were washed twice with PBS and fixed with 4% paraformaldehyde (w/v) at room temperature for 1 h. The cells were washed again with PBS, and the nuclei were stained using TOPRO-3 (1:1000; T3605, Invitrogen, Carlsbad, CA, USA) diluted in PBS at room temperature for 30 min. The cells were then mounted on microscope slides (Paul Marienfeld GmbH) with ProLong Gold antifade reagent (Invitrogen) and observed using a confocal laser scanning microscope (Olympus FV-1000 spectral, Tokyo, Japan). The orange fluorescence from the MF-SERS particles_amine_ and the pseudo-blue fluorescence from TOPRO-3 stained nuclei were monitored and merged with the bright field cell images. In addition, SERS signal from the MF-SERS particles_amine_-treated cells mounted on microscope slides were obtained by point-by-point mapping using a 660 nm laser line at the power of 11.8 mW with a 1-µm step size for 1 s.

### Internalization of MF-SERS particles

Fresh cell culture medium (2.5 mL) was prepared in a 5 mL tube (Eppendorf, Hauppauge, NY, USA), containing MDA-MB-231 cells (2.0 × 10^5^) with different amount of MF-SERS particles_amine_ (0.625, 0.375, 0.125, 0.0625 mg/mL). After incubation at 37 °C for 2 h, the cells containing MF-SERS particles were quantified by fluorescence-activated cell sorting (FACS) analysis using a FACS Calibur Flow Cytometer (BD Biosciences, Franklin Lakes, NJ, USA).

### Magnetic isolation and flow cytometry analysis

Fresh cell culture medium (2.5 mL) was prepared in a 5 mL tube (Eppendorf, Hauppauge, NY, USA), containing MDA-MB-231 cells (2.0 × 10^5^) with or without MF-SERS particles_amine_ (20 μL, 0.25 mg/mL). After incubation at 37 °C for 2 h, a strong magnet (4000 gauss) was placed on one side of the tube, followed by a further 2 h incubation at room temperature. The cells submerged at the bottom of the tube were carefully removed, and only the cells attracted to the wall by the magnet were collected and quantified by fluorescence-activated cell sorting (FACS) analysis using a FACSCalibur Flow Cytometer (BD Biosciences, Franklin Lakes, NJ, USA).

### Antibody-specific binding of MF-SERS particles on MDA-MB-231 cells

The specific binding of the MF-SERS particles_Ab_ to antigen-expressing cells was monitored by observing orange fluorescence of the MF-SERS particles_Ab_ on the cells using a confocal microscope. The CD44-positive (+) cell line (MDA-MB-231 cells) and the CD44-negative (−) cell line (HepG2 cells) were grown in the wells of a 12 well plate with cover glass at a density of 1.0 × 10^6^ cells. The cells were fixed with 4% paraformaldehyde at room temperature for 1 h and blocked with 3% BSA. After washing three times with PBS, 1 mL of PBS containing MF-SERS particles_Ab_ (0.25 mg/mL) or MF-SERS particles (0.25 mg/mL) were added to the cells and incubated at 4 °C for 2 h. The primary CD44 antibody (1:1000 dilution) and fluorescence-conjugated secondary antibodies (1:1000; Alexa Fluor 488) were also treated to MDA-MB-231 cells or HepG2 cells at 4 °C for 2 h as a control (Fig. S9). The cells were washed three times with PBS and visualized using a confocal microscope after nuclei staining as mentioned above.

## Electronic supplementary material

Supplementary Information

Supplementary Movie 1

Supplementary Movie 2

Supplementary Movie 3
