# Supplementary material for: Assembly of Plasmonic and Magnetic Nanoparticles with Fluorescent Silica Shell Layer for Tri-functional SERS-Magnetic-Fluorescence Probes and Its Bioapplications
Source: Sci Rep. 2018 Sep 17;8:13938. doi: 10.1038/s41598-018-32044-7 (PMC6141549; doi:10.1038/s41598-018-32044-7)
Supplement: Supplementary file 1 — Supplementary Information [file 41598_2018_32044_MOESM1_ESM.docx]

**Supplementary information**

**Assembly of Plasmonic and Magnetic Nanoparticles with Fluorescent Silica Shell Layer for Tri-functional SERS-Magnetic-Fluorescence Probes and Its Bioapplications**

Hyung-Mo Kim,^1^‡ Dong-Min Kim,^1^‡ Cheolhwan Jeong,^2^‡ So Yeon Park,^2^ Myeong Geun Cha,^3^ Yuna Ha,^1^ Dahye Jang,^1^ San Kyeong,^2^ Xuan-Hung Pham,^1^ Eunil Hahm,^1^ Sang Hun Lee,^2^ Dae Hong Jeong,^3^ Yoon-Sik Lee,^2^* Dong-Eun Kim,^1^* and Bong-Hyun Jun^1^*

^1^ Department of Bioscience and Biotechnology, Konkuk University, Seoul 05029, Republic of Korea

^2^ School of Chemical and Biological Engineering, Seoul National University, Seoul 08826, Republic of Korea

^3^ Department of Chemistry Education, Seoul National University, Seoul 08826, Republic of Korea

‡ These authors contributed equally to the work.

**Name of Corresponding Authors:** Yoon-Sik Lee, Ph D, Dong-Eun Kim, Ph D, Bong-Hyun Jun, Ph.D

**E-mail:** yslee@snu.ac.kr (Y.-S. Lee), kimde@konkuk.ac.kr (D.-E. Kim), bjun@konkuk.ac.kr (B.-H. Jun)

**Experimental details**

**Counting of Fe_3_O_4_ NPs on the silica surface.** We merged the several TEM images of M-SiO_2_ NPs, and adjusted contrast, brightness and size of TEM images for confirmation Fe_3_O_4_ NPs on the silica surface (Figure S4). After that, we counted the number of Fe_3_O_4_ NPs in front side of NPs in TEM images, and multiplied by 2 to calculate the whole number.

**Cytotoxicity study.** We carried out a cell viability assay using WST-1 reagent (EZ-Cytox Cell Viability Assay Kit, Daeil Lab Service Co Ltd., Seoul, Korea). To determine the cell cytotoxicity of MF-SERS particles at various concentrations, the MDA-MB-231 cells were seeded onto a 96-well plate at a density of 5.0 × 10^3^ cells. After 24 h incubation at 37 °C, the plate was blocked with 3% bovine serum albumin in PBS for 30 min. After washing with PBS twice, 100 µL of media containing 0.1 ~ 10 µg/mL of MF-SERS particles were added to the cells and incubated at 37 °C for 2 hours. To assess cell viability, the absorbance at 450 nm was measured by a VICTOR X3 micro plate reader (PerkinElmer, Waltham, MA, USA).


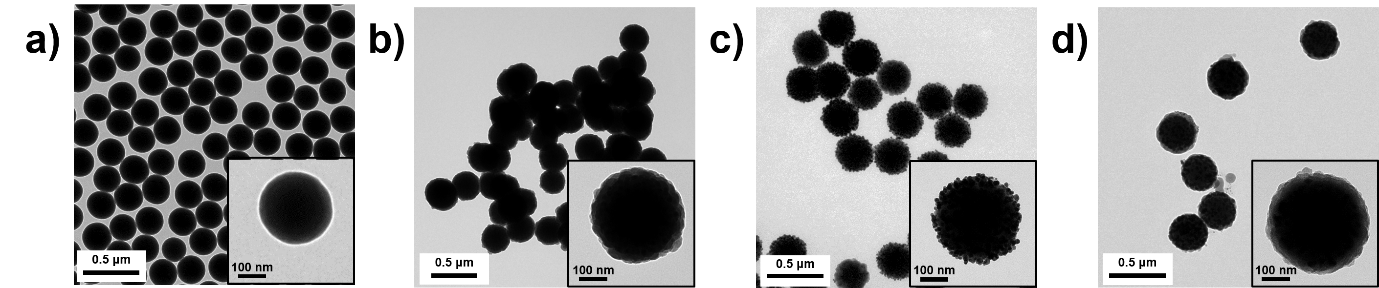


**Figure S1.** TEM analysis Low magnification of a) Silica NPs, b) M-SiO_2_ NPs (Merge), c) Ag-M-SiO_2_ NP, and d) MF-SERS particles.


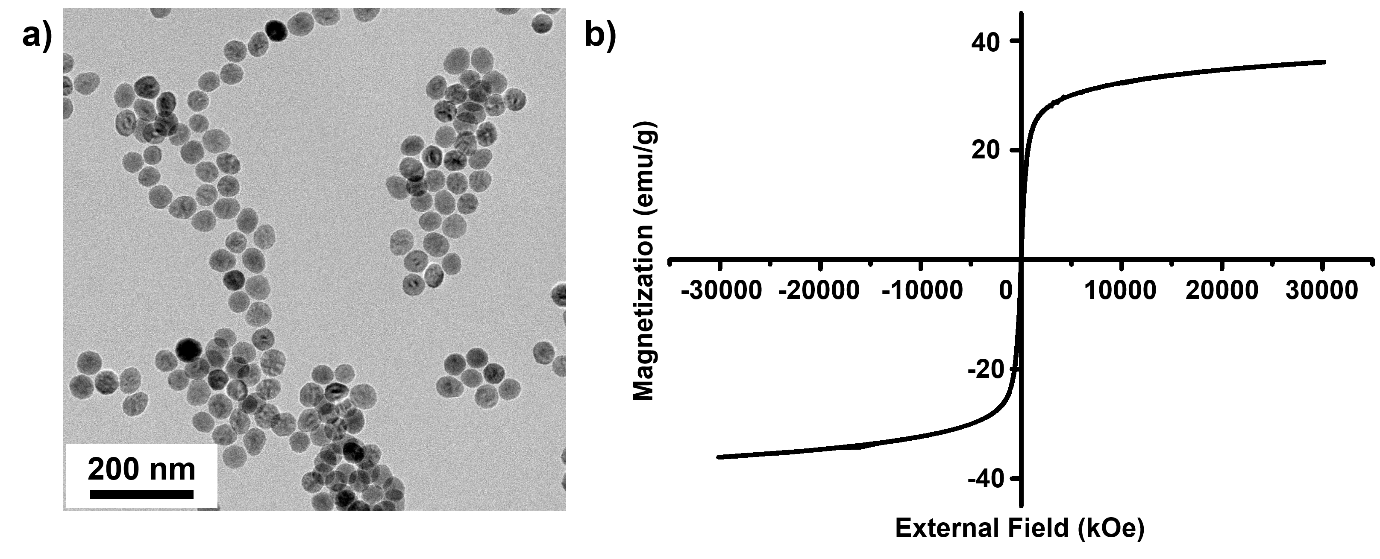


**Figure S2.** Characterization of oleate-coated Fe_3_O_4_ NPs. a) TEM image, b) Hysteresis loop of oleate-coated Fe_3_O_4_ NPs.


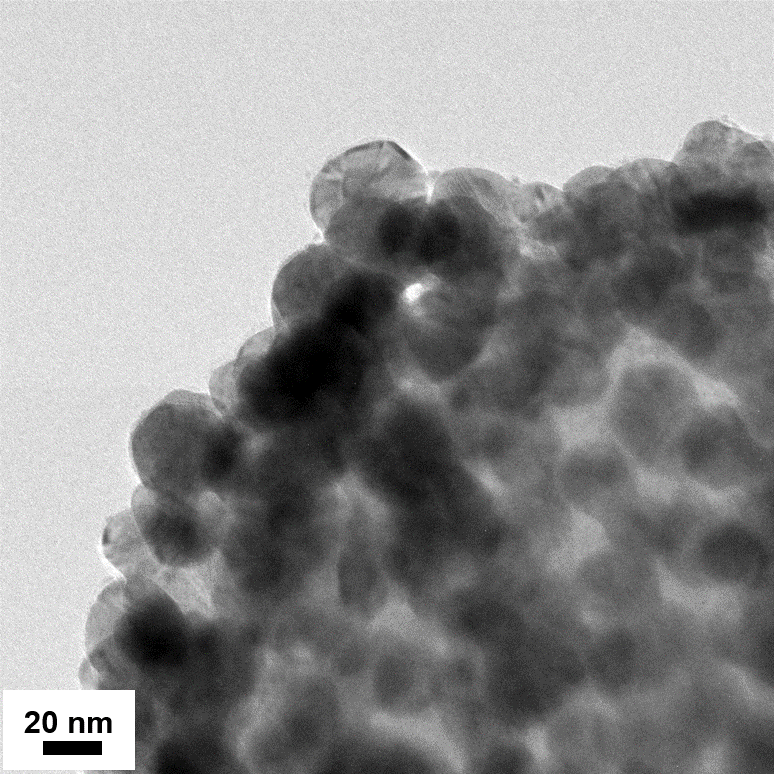


**Figure S3.** High-resolution transmission electron microscopic (HR-TEM) analysis of surface on the Ag-M-SiO_2_ particle.


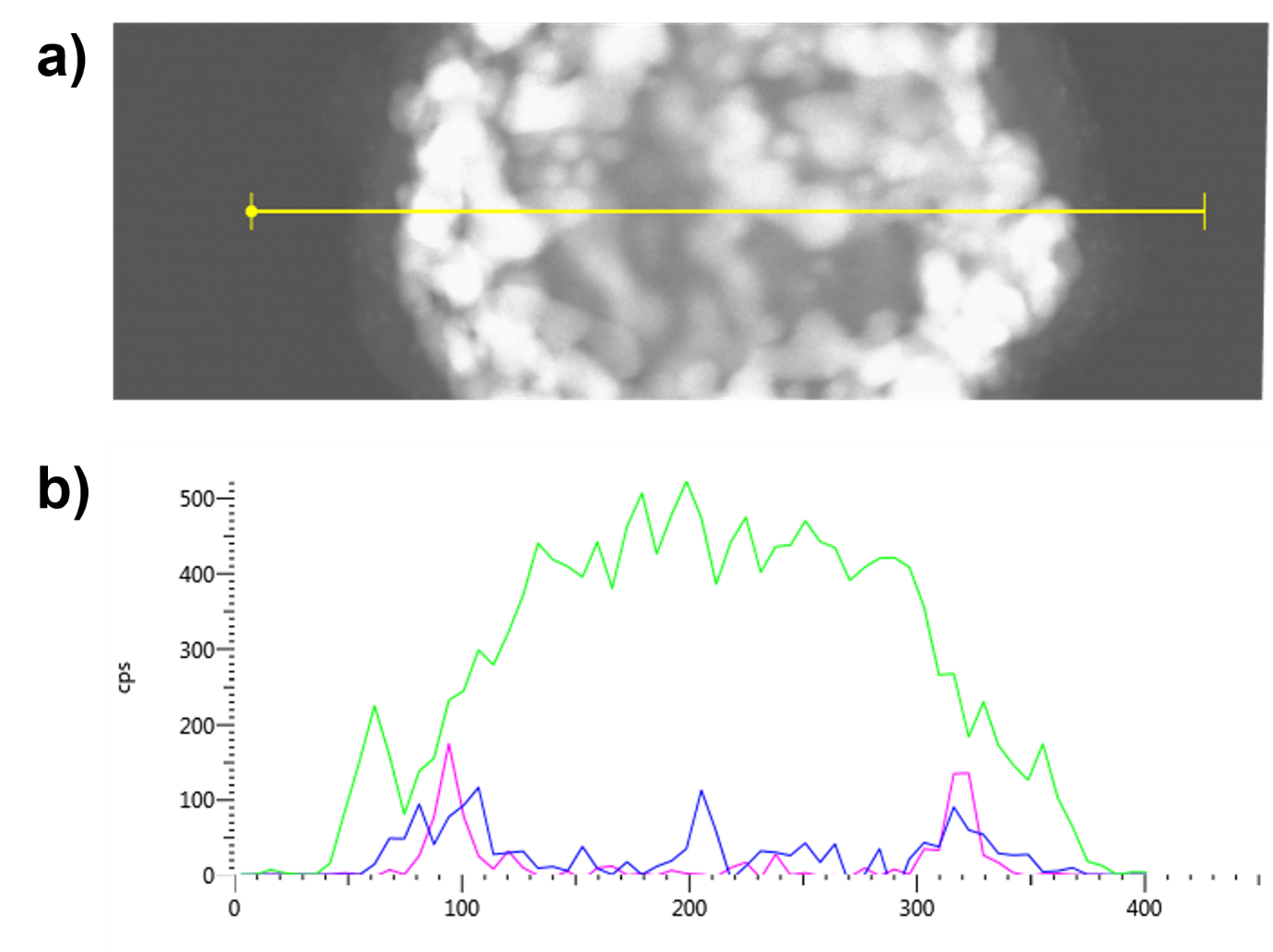


**Figure S4.** a) Scanning transmission electron microscopy analysis and b) graph of size for each element in MF-SERS particle (green: Si, blue: Ag, purple: Fe).


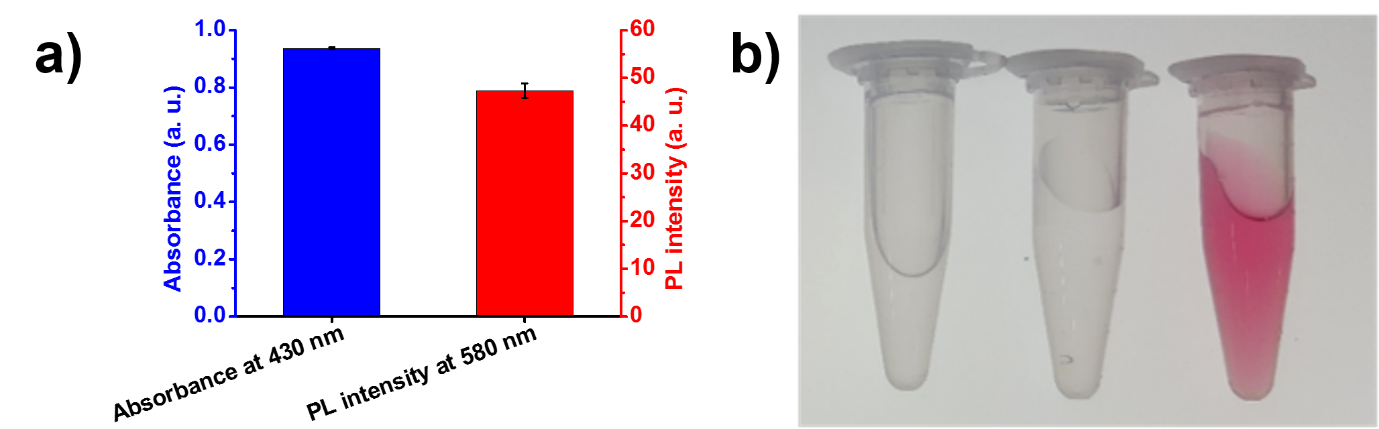


**Figure S5.** Various tests of synthesized MF-SERS particles. a) reproducibility, b) storage (EtOH (Left), PBS (pH 7.4, Middle) and cell culture media (Right)).


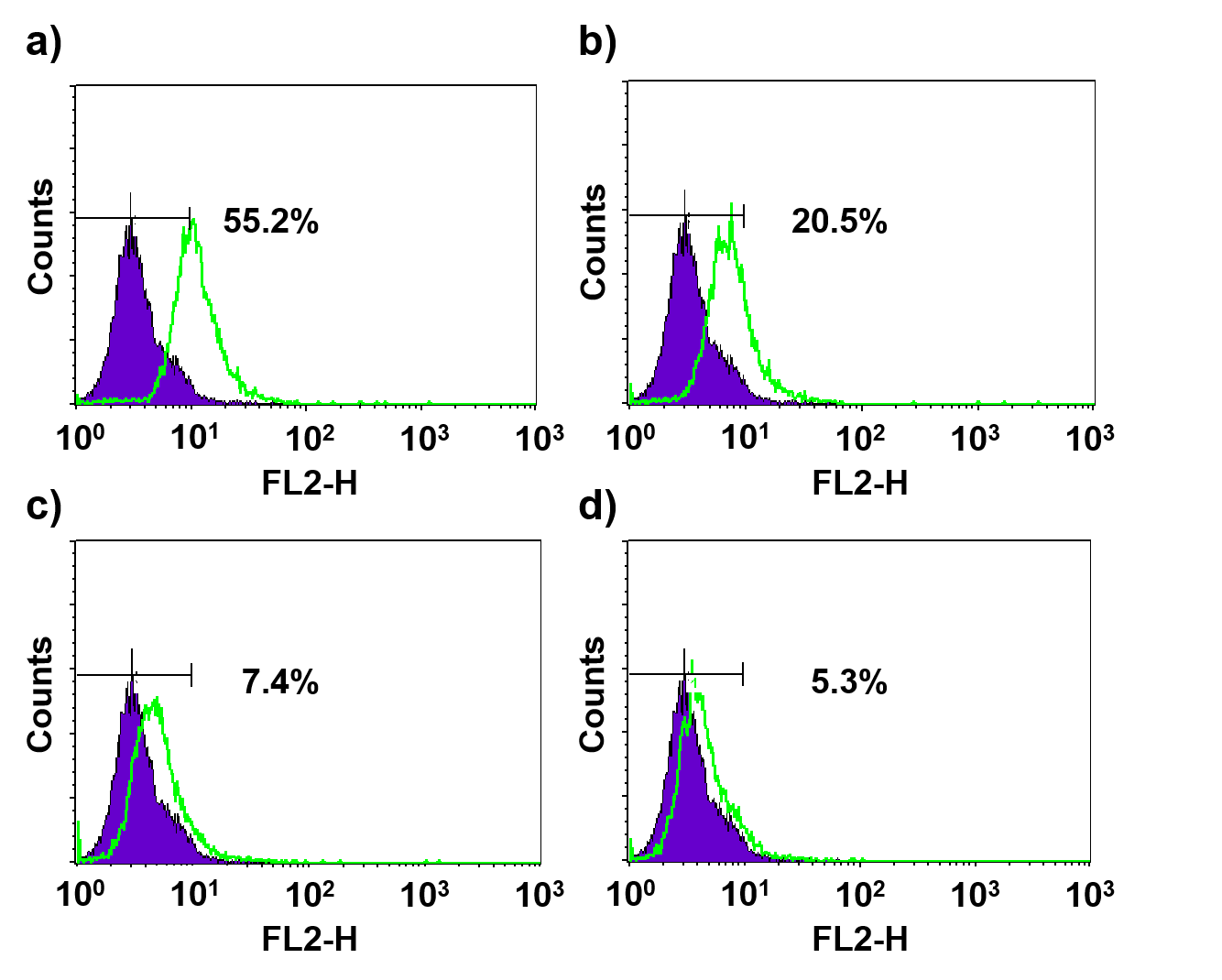


**Figure S6.** Flow cytometric measurement of RITC labeled MF-SERS particles_amine_ uptake by MDA-MB-231 cells after being incubated with a) 0.625 mg/mL, b) 0.375 mg/mL, c) 0.125 mg/mL or d) 0.0625 mg/mL of MF-SERS particles_amine_ for 2 h. The background auto fluorescence of untreated cells is shown as a control (black line graph filled with purple). The population of uptake cells correlated with the amount of SERS particles treated.

**
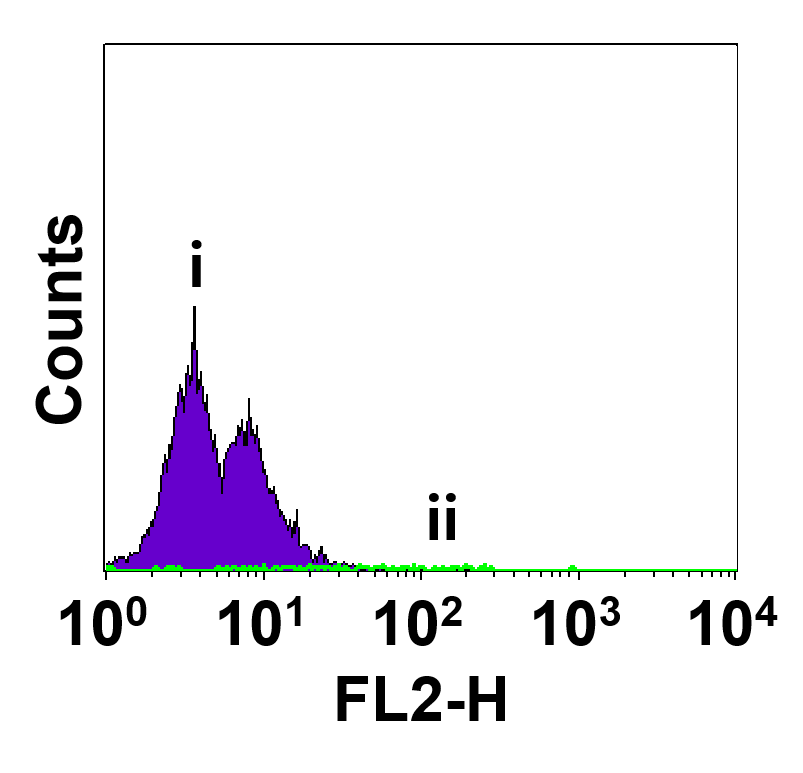
**

**Figure S7.** Flow cytometry analysis of MDA-MB-231 cells (i) and only MF-SERS particles (ii).


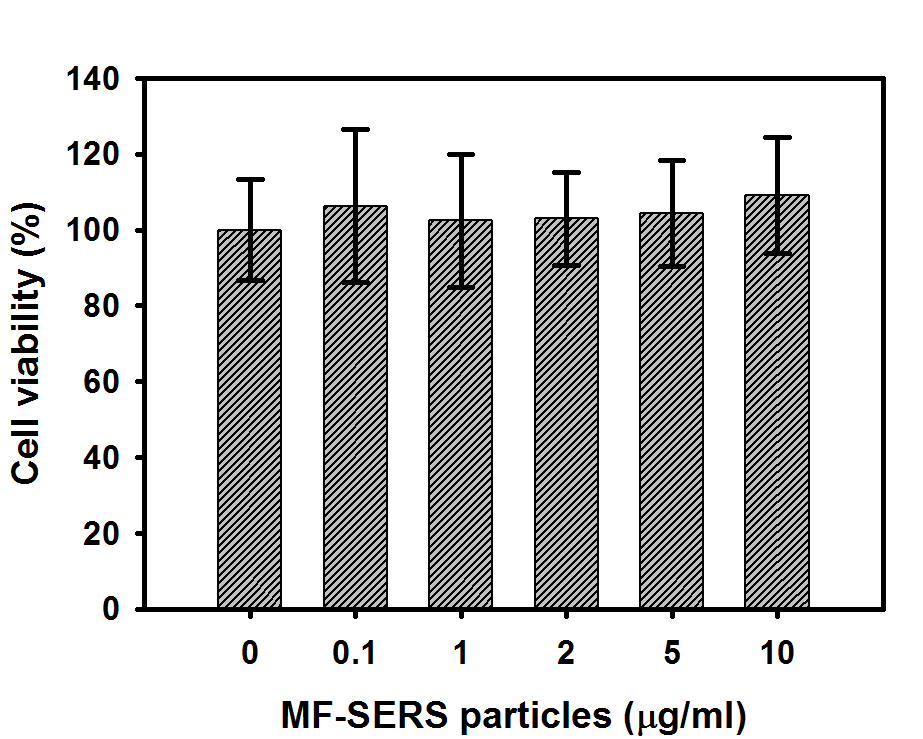


**Figure S8.** The cytotoxicity of MF-SERS particles used to treat MDA-MB-231 cells. The cell viability was measured using the WST-1 assay after the cells were treated with MF-SERS particles (0 ~ 10 µg/mL) for 2 hours.

**
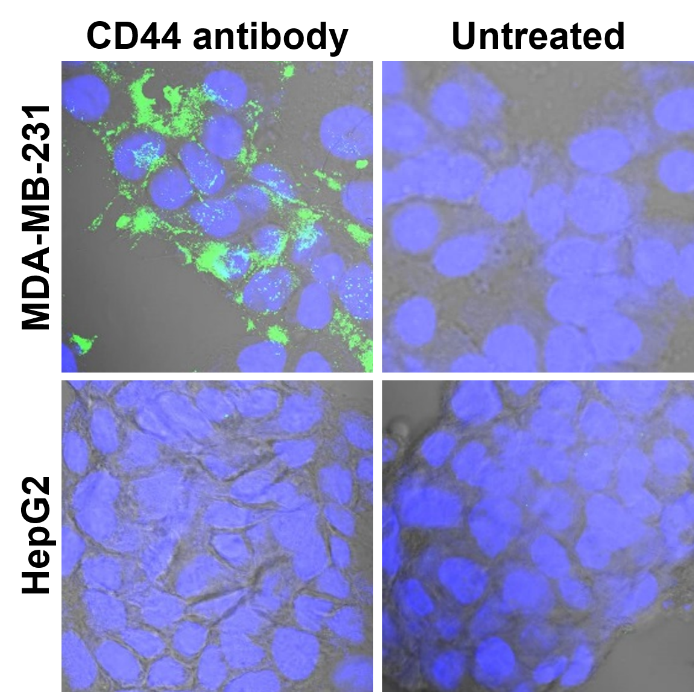
**

**Figure S9.** Confocal fluorescence microscopy images of MDA-MB-231 cells treated with CD44 antibodies or untreated MDA-MB-231 cells, and HepG2 cells treated with CD44 antibodies or untreated HepG2 cells at 4 °C for 2 h. The green fluorescence represents the expression of CD44 antigen, pseudo-blue fluorescence shows the nuclei, and the bright field images of cells show the cell boundary.


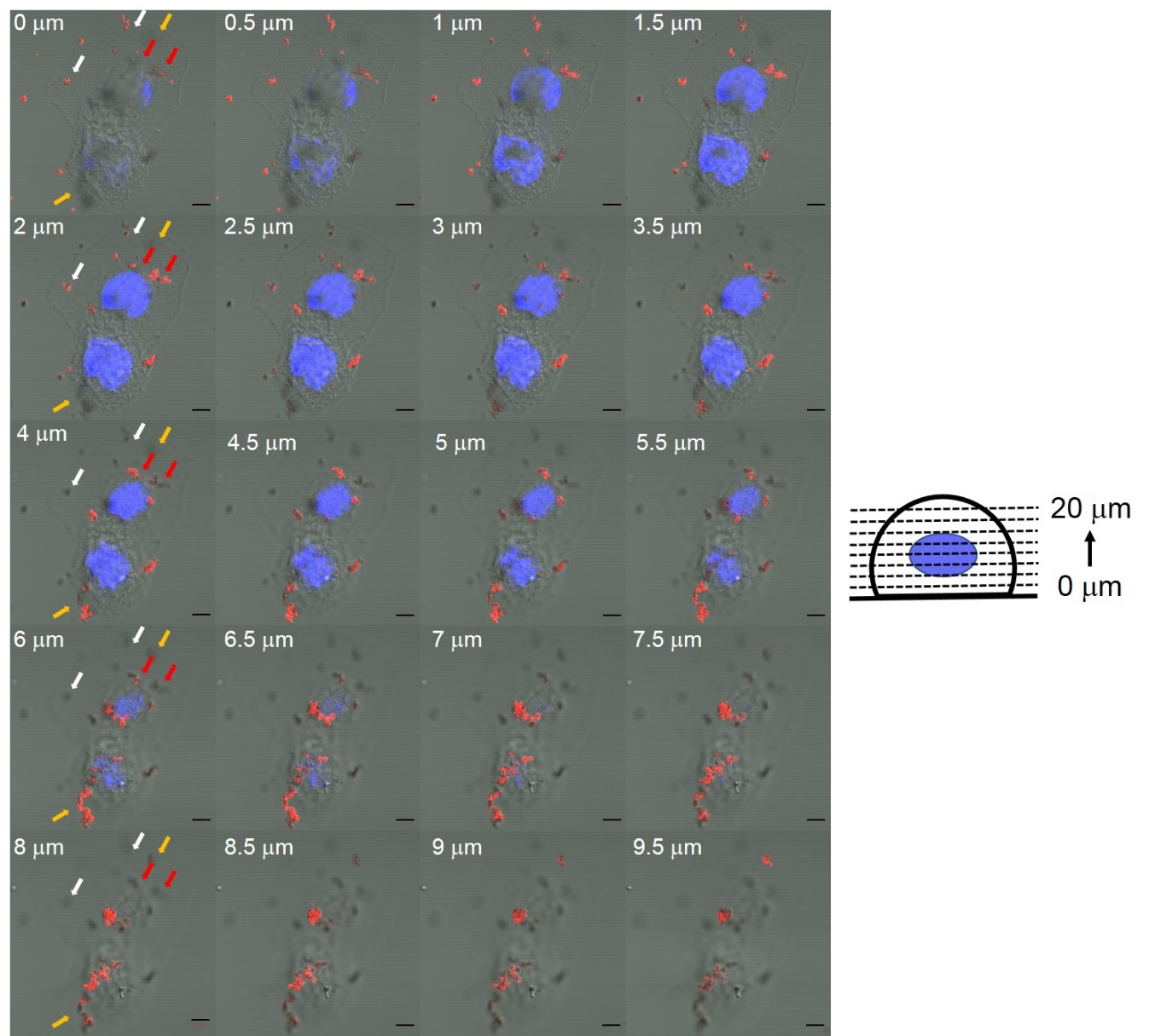


**Figure S10.** Confocal fluorescence microscopy images of MDA-MB-231 cells treated with MF-SERS particles_Ab_ with different Z-axis stacks. Fluorescent cell images of 20 stacks of Z-axis sections were displayed, ranging from 0 to 9.5 µm (each stack with 0.5 μm height) using the confocal Z-stack acquisition software after incubation the cells with MF-SERS particles_Ab_ for 2 h. The pseudo-red fluorescence and the pseudo-blue fluorescence represent MF-SERS particles_Ab_ and nuclei stained by TOPRO-3, respectively. The fluorescence images were merged with the bright field cell image. Red fluorescence (shown by red arrows) represents MF-SERS particles_Ab_ that are readily internalized in the cells. Red fluorescent spots shown at the bottom were disappeared as the height of Z-stack increases (white arrows), while some red fluorescent spots were only observed at the top Z-stacks (yellow arrows). The black scale bar represents 5 µm.

**
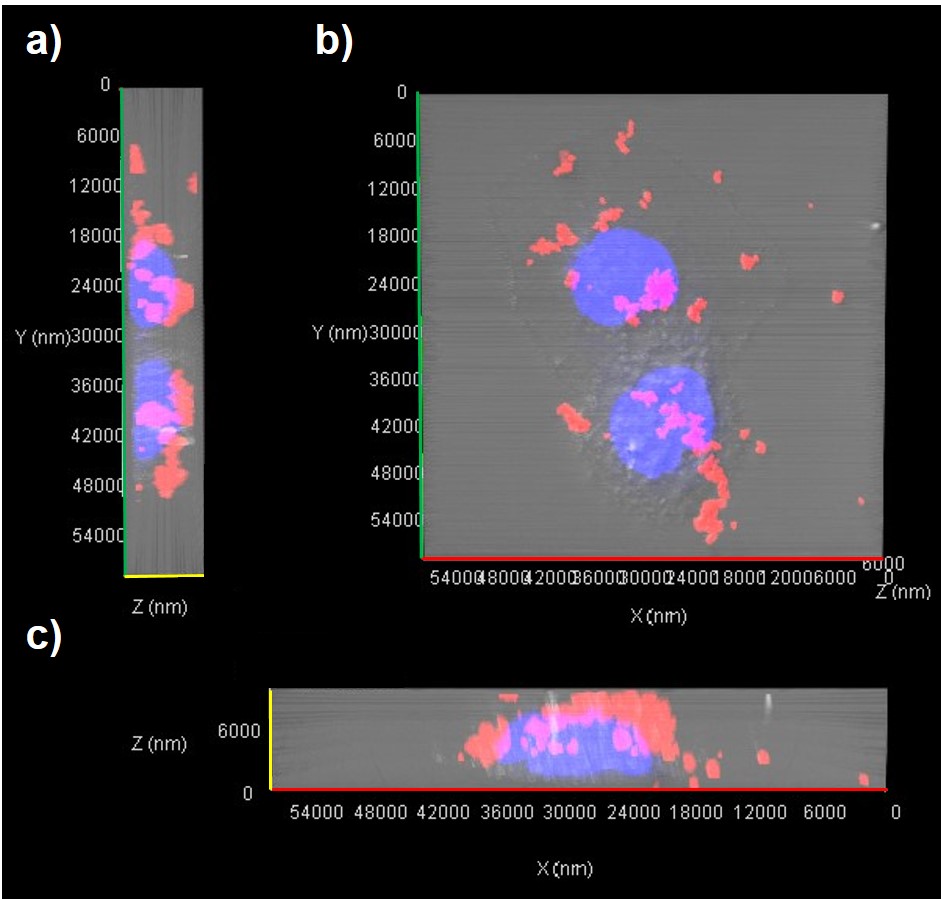
**

**Figure S11.** Orthogonal view images obtained in Fig. S10. Each image shows (a) YZ, (b) XY, and (c) XZ projections. The pseudo-red fluorescence and the pseudo-blue fluorescence represent MF-SERS particles_Ab_ and nuclei stained by TOPRO-3, respectively. The fluorescence images were merged with the bright field cell image.

**Supplementary movie legends:**

**Supplementary Movie 1.** Twenty Z-stack images obtained in Fig. S10 were sequentially visualized using series of Z-stacks ranging from 0 to 9.5 µm.

**Supplementary Movie 2 and 3.** Spatial distribution of the MF-SERS particles_Ab_ fluorescence spots by using 3D image reconstruction of Z-stack images obtained in Fig. S10. Animation of the orthogonal images were displayed by rotating about X axis (Movie S2) and Y axis (Movie S3).
